# Silencing LINC00482 inhibits tumor-associated inflammation and angiogenesis through down-regulation of MMP-15 *via* FOXA1 in bladder cancer

**DOI:** 10.18632/aging.202247

**Published:** 2020-12-11

**Authors:** Yizhuo Wang, Liping Zhang, Na Wei, Yue Sun, Weiyun Pan, Yan Chen

**Affiliations:** 1 Cancer Center, The First Hospital of Jilin University, Changchun 130021, P. R. China; 2 Department of Cardiology, The First Hospital of Jilin University, Changchun 130021, P. R. China; 3 The First Operating Room, The First Hospital of Jilin University, Changchun 130021, P. R. China; 4 Department of Intensive Care Unit (ICU), The First Hospital of Jilin University, Changchun 130021, P. R. China; 5 Department of Gastrointestinal Surgery, The First Hospital of Jilin University, Changchun 130021, P. R. China

**Keywords:** LINC00482, bladder cancer, FOXA1, MMP15, inflammation

## Abstract

Multiple studies have previously demonstrated that long intergenic non-coding RNAs (lincRNAs) play an important role in the development of bladder cancer. However, little is known regarding the underlying molecular mechanisms of LINC00482 functions in bladder cancer. The current study aimed to elucidate the role of LINC00482 in the progression of bladder cancer. The initial step was to detect the expressions of LINC00482 and MMP15 in bladder cancer cells and tissue. According to the results from the RT-qPCR, LINC00482 and MMP15 were both highly expressed in bladder cancer cells and tissue. The relationship among LINC00482, FOXA1 and MMP15 was studied *via* dual-luciferase reporter assay. LINC00482 was positively correlated with MMP15. LINC00482 promoted MMP15 expression by recruiting FOXA1. Using the gain- and loss-of-function approaches, silencing of LINC00482 resulted in the downregulation of VEGF and NF-κB protein levels, decreased expression of inflammatory factors, and inhibited angiogenesis. Silencing of LINC00482 also suppressed tumor-associated inflammation and angiogenesis *in vivo*, which was found to be reversed by the overexpression of MMP15. The present study demonstrated that LINC00482 induced the expression of MMP15 by interacting with FOXA1, thereby contributing to the inflammation and angiogenesis in bladder cancer.

## INTRODUCTION

Bladder cancer is one of the most prevalent cancers worldwide and develops through two distinct pathways, papillary and nonpapillary based on the clinical forms of the primary tumors [1]. In 2018, there were approximately 549,000 new cases and 200,000 deaths of bladder cancer globally. The incidence of bladder cancer was higher in males, with morbidity and mortality rates about four times than that in women [2]. Bladder cancer is characterized by the clinical manifestations of painless hematuria (the most common symptom), dysuria, as well as metastases [3]. As an exceedingly prevalent disease, the risk factors of bladder cancer mainly include smoking, occupational exposure, inflammation, radiation and chemotherapy [4, 5]. Bladder cancer has a high incidence of recurrence, particularly among the people in developed countries [6]. Hence, it is essential to investigate the molecular mechanisms underlying the progression of bladder cancer, which might provide a novel therapeutic strategy for bladder cancer treatment.

Long non-coding RNAs (lncRNAs), are clinically important regulatory players and novel biomarkers in cancer, and have been demonstrated to be expressed abnormally in a variety of cancers hence playing important roles in driving and maintaining the occurrence, development, and progression of tumors [7]. Cumulative data from a variety of studies demonstrates that lncRNAs such as H19, MALAT-1, and AC114812.8 contribute to the acceleration of the bladder cancer progression [8–10]. However, no data is currently available regarding the biological roles of LINC00482 in bladder cancer. Dual-luciferase reporter gene assay analysis revealed that LINC00482 could positively regulate the expression of matrix metalloproteinase15 (MMP15). MMP15, also known as MT2-MMP, has been identified from a human lung cDNA library. During progression of cancer, downregulation of MMP15 expression exerted inhibitory functions in tumor growth [11]. Previously conducted studies have also reported that site 2 (TGTATTCTTTG) of MMP15 promoter region was the binding site of transcription factor forkhead box A1 (FOXA1). FOXA1 is a pioneer transcription modulator and the down-regulation of FOXA1 is implicated as an independent indicator of reduced overall survival in humans with bladder cancer [12, 13]. However, the underlying molecular mechanisms by which LINC00482 functions in bladder cancer remain elusive. In the current study, *in vitro* and *in vivo* experiments were performed to investigate the specific role of LINC00482 in bladder cancer. With gain- and loss-function methods, LINC00482 was transfected into cells and injected into mice to observe the alteration of related indexes.

## RESULTS

### The significance of LINC00482 in bladder cancer

**Figure 1:**
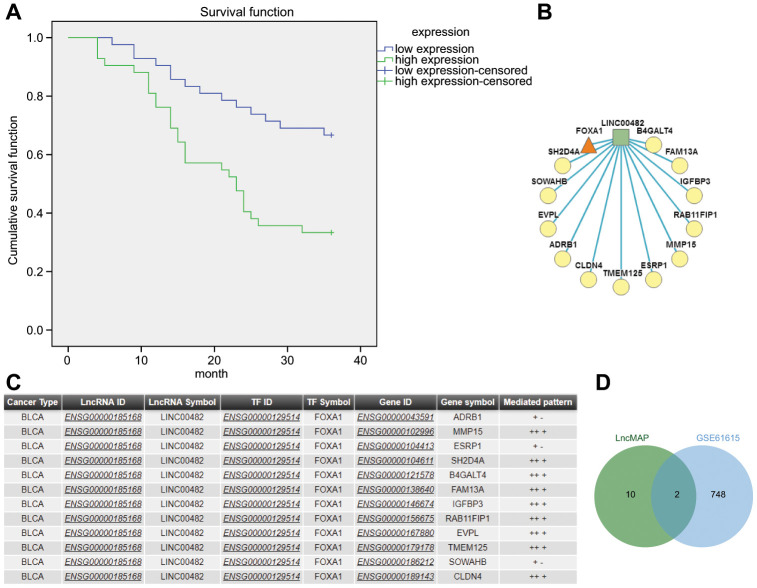
**The significance of LINC00482 in bladder cancer.** (**A**) Kaplan-Meier analysis of LINC00482 and survival of bladder cancer patients; (**B**) the LncRNA-TF-Gene Network obtained by analyzing the triplet formed by LINC00482 and FOXA1 on LncMAP, where the square represented LncRNA, the triangle represented TF and the circle represented gene; (**C**) the relevant information obtained by analyzing the triplet formed by LINC00482 and FOXA1 on LncMAP; (**D**) the intersection analysis of genes in triplet and differential genes in GSE61615 on jvenn.

A comprehensive analysis of bladder cancer microarray data GSE61615 revealed 4 significant differential LincRNAs, LINC00051, LINC00116, LINC00338 and LINC00482, where logFC of LINC00482 was the highest (Table 1). The GSE61615 analysis further demonstrated that LINC00482 had the most significant change and was up-regulated in bladder cancer, hence it was selected as the candidate LincRNA for subsequent experiments. Furthermore, the Kaplan-Meier analysis revealed better prognosis occurred in patients with low expressions of LINC00482 (Figure 1A).

**Table 1:** Expression of LINCRNA in bladder cancer-related microarray data GSE61615.

	**logFC**	**AveExpr**	**t**	**P.Value**	**adj.P.Val**	**B**
LINC00051	-1.532760888	4.50743302	-2.684527995	0.04950967	0.8936813	-4.536943565
LINC00116	-1.357142063	2.01012801	-2.731014197	0.04703172	0.8936813	-4.535845581
LINC00338	1.3434562630	6.96320616	4.0099925210	0.01311827	0.8936813	-4.514431177
LINC00482	1.7035580500	9.78930641	2.7372055400	0.04671240	0.8936813	-4.535701429

Note: LogFC: Logarithm fold change. AveExpr: average expression across all samples. t: logFC divided by its standard error (based on t) from test. P Value: Raw p-value. adj.P.Val: Benjamini-Hochberg false discovery rate adjusted p-value. B: log-odds that the gene is differentially expressed.

The LncMAP analysis determined that LINC00482 formed lncRNA-TF-gene triplet in bladder cancer, among which FOXA1 formed 12 triplets, only secondary to SMARCC2 which formed 19 triplets but it has never been indicated to be associated with bladder cancer. Moreover, FOXA1 was indicated to be the independent predictor of poor prognosis of bladder cancer and also a marker of luminal and basal subtypes in bladder cancer [13, 14]. Therefore, FOXA1 was selected as a candidate transcription factor for further experimentation. The intersection between differentially expressed genes in the triplets formed by LINC00482 and FOXA1 (Figure 1B, 1C) and in GSE61615 microarray data was analyzed together to obtain MMP15 and ESRP1 as the candidate factors (Figure 1D). The TF-gene rewiring score of MMP15 (9.99e-01) was revealed to be larger than that of ESRP1 (9.97e-01), therefore MMP15 was selected as a candidate gene for further experimentation. Based on the above-mentioned evidence, it was hypothesized that LINC00482 inhibited progression of bladder cancer through regulating MMP-15 *via* FOXA.

### LINC00482 is highly expressed in bladder cancer tissues and cells

**Figure 2:**
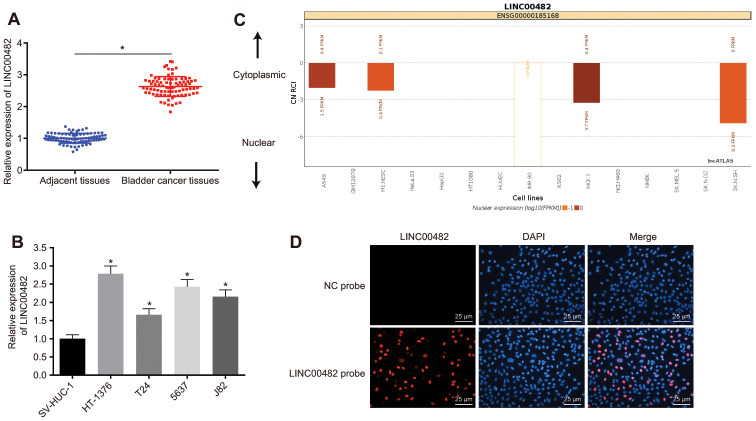
**LINC00482 is highly expressed in bladder cancer tissues and cells.** (**A**) The comparison of LINC00482 expression between the bladder cancer and adjacent normal tissues detected by RT-qPCR, n = 84, * *p* < 0.05 *vs*. adjacent normal tissues; (**B**) the expression of LINC00482 in 4 human bladder cancer cell lines and human normal bladder epithelial cells (SV-HUC-1) detected by RT-qPCR, * *p* < 0.05 *vs.* SV-HUC-1 cells; (**C**) the localization of LINC00482 in different cells by online analysis; (**D**) subcellular localization of LINC00482 detected by RNA-FISH (× 400). The measurement data were presented as mean ± standard deviations. Paired *t*-test was used for intra-group comparison, while comparisons among multiple groups were analyzed by one-way analysis of variance, followed by Tukey’s post-hoc test. Experiments were repeated three times.

With the aim to confirm the expression of LINC00482 in cells and clinical tissues, RT-qPCR analysis was performed on 84 bladder cancer tissue and cell samples. The analysis revealed that LINC00482 was significantly upregulated in human bladder cancer tissues, when compared to adjacent tissues (*p* < 0.05) (Figure 2A). There was a significant statistical difference between LINC00482 expression at the T stage or differential grade, but no significant statistical difference between LINC00482 expression and age, gender, and lymphatic metastasis (Table 2). Furthermore, LINC00482 was also highly expressed in HT-1376, T24, 5637(HTB-9), and J82 cells (*p* < 0.05), in comparison to SV-HUC-1 cells, with the highest expression noted in the HT-1376 cells (Figure 2B). The localization of LINC00482 in different cells was analyzed online (Figure 2C). In human bladder cancer cell line HT-1376, LINC00482 was mainly found to be localized in the nucleus, according to the results from RNA-fluorescence *in situ* hybridization (FISH) assay (Figure 2D).

**Table 2:** Patient's clinical data.

	**Case**	**LINC00482**		**P.Value**
		**High**	**Low**	
Age				0.811
<60	25	12	13	
≥60	59	30	29	
Gender		0.643
Male	56	27	29	
Female	28	15	13	
T stage				<0.001*
pTa-T1	28	3	25	
pT2-T4	56	39	17	
Grade				<0.001*
Low	43	38	19	
High	41	4	37	
Lymph node metastasis				0.127
No	43	25	18	
Yes	41	17	24	

### Silencing LINC00482 inhibits inflammation and angiogenesis

**Figure 3:**
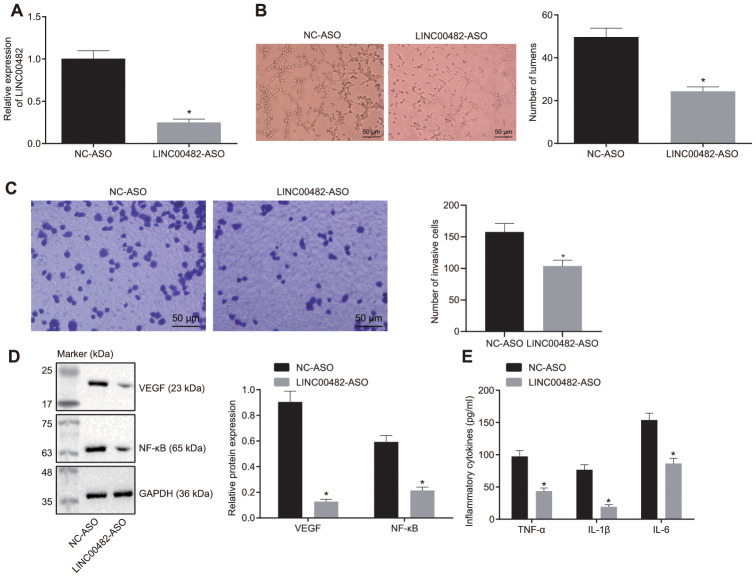
**Silencing LINC00482 inhibits inflammation and angiogenesis.** (**A**) The silencing efficiency of LINC00482 analysis by RT-qPCR; (**B**) The tube formation ability after treatment of LINC00482-ASO (× 200); (**C**) Representative images of Transwell assay and quantification of migratory cells upon treatment of LINC00482-ASO and NC-ASO (× 200); (**D**) the expression of VEGF and NF-κB detected by Western blot analysis; (**E**) the levels of TNF-α, IL-1β, IL-6 tested by ELISA. The measurement data were presented as mean ± standard deviations. The differences between two groups were compared by unpaired t test. Experiments were repeated three times; * *p* < 0.05 *vs*. cells treated with NC-ASO.

The upregulation of LINC00482 in bladder cancer tissue and cells was confirmed by the above experiments. The effect of LINC00482 on inflammation and angiogenesis was put under further investigation. The results from the RT-qPCR analysis confirmed that treatment of LINC00482-ASO effectively silenced expression of LINC00482 (*p* < 0.05) (Figure 3A). Furthermore, the tube formation experiment revealed the reduction of number of tubes in cancer cells following LINC00482-ASO treatment when compared to the NC-ASO control (*p* < 0.05) (Figure 3B). Whereas, LINC00482-ASO treatment declined the percentage of invading cells, suppressing the invasion of bladder cancer cells (Figure 3C). Moreover, the Western blot analysis and enzyme-linked immunosorbent assay (ELISA) were performed to detect the expression of inflammatory factors upon treatments. The results of these analyses established that the silencing of LINC00482 significantly decreased expression of VEGF and NF-κB and levels of TNF-α, IL-1β, and IL-6 (*p* < 0.05) (Figure 3D, 3E). Cumulative data from the aforementioned experiments indicated that silencing LINC00482 could inhibit cancer cell invasion, inflammation and angiogenesis in bladder cancer.

### LINC00482 up-regulates the expression of MMP15 by recruiting FOXA1

**Figure 4:**
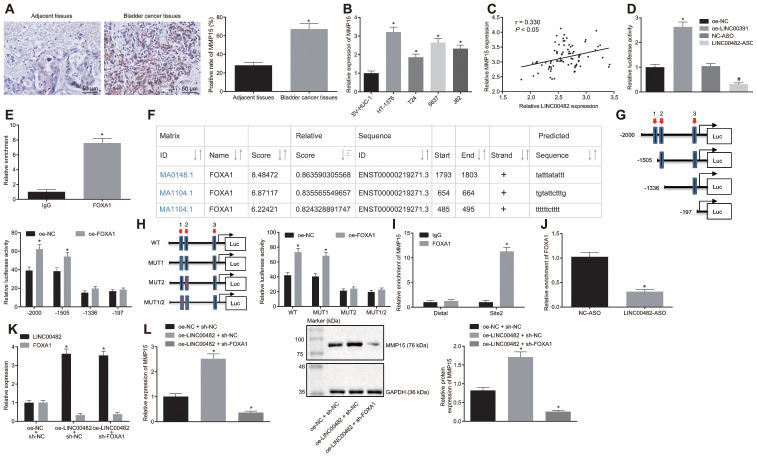
**LINC00482 up-regulates the expression of MMP15 by recruiting FOXA1.** (**A**) MMP15 expression in bladder cancer and adjacent normal tissues detected by immunohistochemistry (× 200), * *p* < 0.05 *vs*. adjacent normal tissues; (**B**) Expression of MMP15 in 4 kinds of bladder cancer cells and SV-HUC-1 cell detected by RT-qPCR; * *p* < 0.05 *vs*. SV-HUC-1 cell; (**C**) The correlation between LINC00482 and MMP15 expression in the bladder cancer tissues analyzed by Pearson correlation coefficient; (**D**) Dual-luciferase reporter gene assay was used to study the effect of LINC00482 on the activity of MMP15 promoter, * *p* < 0.05 *vs.* the oe-NC group, # *p* < 0.05 *vs*. the NC-ASO group; (**E**) The interaction between LINC00482 and FOXA1 verified by RIP assay, * *p* < 0.05 *vs*. the IgG group; (**F**) FOXA1 protein might combine the three sites of MMP15 promoter region by online analysis; (**G**) The truncated MMP15 recombinant luciferase reporter vector co-transfected with FOXA1 expression vector into HT-1376 cells for dual-luciferase reporter assay, * *p* < 0.05 *vs*. the oe-NC group; (**H**) the mutated MMP15 recombinant luciferase reporter vector co-transfected with FOXA1 expression vector into HT-1376 cells for dual-luciferase reporter assay, * *p* < 0.05 *vs.* the oe-NC group; (**I**) Enrichment of FOXA1 at site 2 in MMP15 promoter region analyzed by ChIP assay, * *p* < 0.05 *vs.* the IgG group; (**J**) Enrichment FOXA1 on MMP15 after silencing LINC00482 in HT-1376 cells detected by ChIP assay, * *p* < 0.05 *vs*. the NC-ASO group; (**K**) Transfection efficiency of LINC00482 and FOXA1 in each group by RT-qPCR, * *p* < 0.05 *vs.* cells treated with oe-NC + sh-NC; (**L**) The expression of MMP15 after treatment of oe-LINC00482 and sh-FOXA1 detected by RT-qPCR and Western blot analysis, * *p* < 0.05 *vs.* cells treated with oe-NC + sh-NC. The measurement data were presented as mean ± standard deviations. Paired t-test was used for intra-group comparison, while differences between two groups were compared by unpaired t test. Comparisons among multiple groups were analyzed by one-way analysis of variance, followed by Tukey’s post-hoc test. Experiments were repeated three times.

Subsequently, the relationship among LINC00482, FOXA1 and MMP15 was evaluated. The expression of MMP15 in bladder cancer tissues and cells was detected by immunohistochemistry and the RT-qPCR analysis. They showed that MMP15 expression was significantly higher in bladder cancer tissues and cells (all *p* < 0.05) (Figure 4A, 4B). Furthermore, LINC00482 was indicated to positively correlate with MMP15 expression in bladder cancer tissues (*p* < 0.05) (Figure 4C), when the correlation analysis between LINC00482 and MMP15 expression was carried out on the 84 paired bladder cancer tissues.

To explore the interaction among the three factors, based on the results from bioinformatics and online databases, the dual-luciferase reporter gene assay analysis was performed. Oe-NC, oe-LINC00482, NC-ASO, and LINC00482-ASO were co-transfected with MMP15-2Kb luciferase reporter vector, respectively. The results showed that the MMP15 promoter activity of the oe-LINC00482 group was significantly higher than that of the oe-NC group (*p* < 0.05), meanwhile the MMP15 promoter activity of the LINC00482-ASO group was found to be reduced (*p* < 0.05) (Figure 4D). Aforementioned data indicated that LINC00482 could positively regulate the expression of MMP15. Moreover, the immunoprecipitation (RIP) assay was conducted to verify whether LINC00482 could interact with transcription factor FOXA1. The results established that the FOXA1 binding to LINC00482 was significantly increased in comparison to the IgG control (*p* < 0.05), indicating that FOXA1 protein could bind to LINC00482 (Figure 4E). Additionally, as revealed by UCSC (http://genome.ucsc.edu/) and JASPAR (http://jaspar.genereg.net/) online analyses, the FOXA1 protein may have three potential binding sites on the MMP15 promoter region (Figure 4F). To identify the specific binding sites, the dual-luciferase reporter gene assay was performed. The truncated or mutated MMP15 recombinant luciferase reporter system was co-transfected with FOXA1 expression vector into HT-1376 cells (Figure 4G, 4H). The results of the dual-luciferase reporter gene assay revealed that site 2 (TGTATTCTTTG) was the specific site of FOXA1 protein binding to MMP15 promoter region. Furthermore, the Chromatin immunoprecipitation (ChIP) assay confirmed the previous results that (Figure 4I), amplification of primers at site 2 of FOXA1 protein binding was remarkably longer than that of Distal primer (*p* < 0.05). In order to further investigate the role of LINC00482 in the regulation of FOXA1 and MMP15, LINC00482 was silenced in HT-1376 cells for ChIP analysis (Figure 4J). Upon treatment with LINC00482-ASO, the amplification products using MMP15 site 2 primer were found to be significantly reduced (*p* < 0.05).

Additionally, to investigate whether LINC00482 promoted MMP15 expression by recruiting FOXA1, cancer cells were transfected with oe-NC + sh-NC, oe-LINC00482 + sh-NC, and oe-LINC00482 + sh-FOXA1. The RT-qPCR analysis confirmed the efficiency of transfection of oe-LINC00482 and sh-FOXA1 (*p* < 0.05) (Figure 4K). Expression of MMP15 was also detected by RT-qPCR and Western blot analysis (Figure 4L). Following the treatment with oe-LINC00482 + sh-NC, MMP15 expression was found to be upregulated (*p* < 0.05), while cells treated with oe-LINC00482 + sh- FOXA1 exhibited a decreased MMP15 expression (*p* < 0.05). This cumulative data demonstrated that LINC00482 could regulate the expression of MMP15 *via* recruiting FOXA1.

### Silencing LINC00482 inhibits inflammation and angiogenesis through down-regulation of MMP-15 *via* recruiting FOXA1

**Figure 5:**
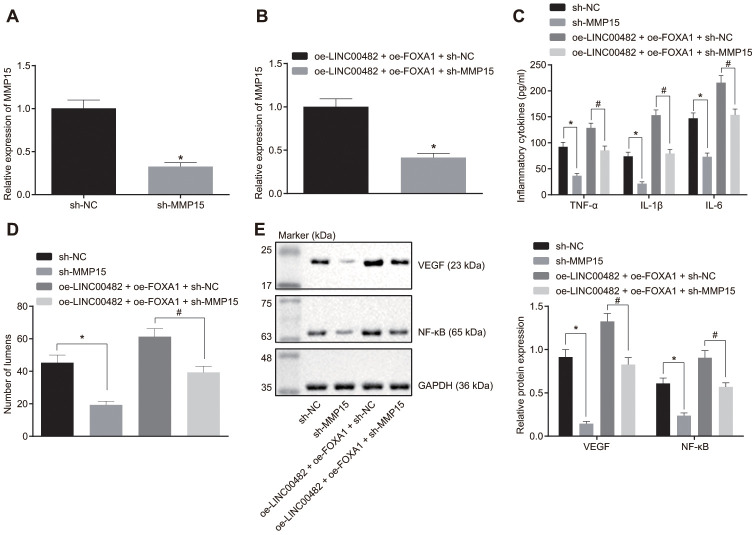
**Silencing LINC00482 inhibits inflammation and angiogenesis through down-regulation of MMP-15 by targeting FOXA1.** (**A**) The interference efficiency of MMP15 confirmed by RT-qPCR after treatment of sh-MMP15, * *p* < 0.05 *vs.* cells treated with sh-NC; (**B**) The interference efficiency of MMP15 confirmed by RT-qPCR after treatment of oe-LINC00482 + oe-FOXA1 + sh-MMP15, * *p* < 0.05 *vs*. cells treated with oe-LINC00482 + oe-FOXA1 + sh-NC; (**C**) Expression levels of TNF-α, IL-1β, IL-6 tested by ELISA, * *p* < 0.05 *vs.* cells treated with sh-NC, # *p* < 0.05 *vs.* cells treated with oe-LINC00482 + oe-FOXA1 + sh-NC; (**D**) The tube formation ability after treatment of sh-MMP15, oe-LINC00482 or oe-FOXA1 in each group, * *p* < 0.05 *vs.* cells treated with sh-NC, # *p* < 0.05 *vs*. cells treated with oe-LINC00482 + oe-FOXA1 + sh-NC; (**E**) Expressions of VEGF and NF-κB detected by Western blot analysis, * *p* < 0.05 *vs.* cells treated with sh-NC, # *p* < 0.05 *vs.* cells treated with oe-LINC00482 + oe-FOXA1 + sh-NC. The measurement data were presented as mean ± standard deviations. The differences between two groups were compared by unpaired t test. Experiments were repeated three times.

The underlying mechanism how LINC00482, FOXA1 and MMP15 function in bladder cancer was further investigated to study the underlying mechanisms, bladder cancer cells were transfected with sh-MMP15, sh-NC, oe-LINC00482 + oe-FOXA1 + sh-MMP15, or oe-LINC00482 + oe-FOXA1 + sh-NC. The interference efficiency of sh-MMP15 was confirmed by the RT-qPCR analysis (Figure 5A, 5B). Furthermore, the results from the ELISA and Western blot analyses depicted that the treatment with sh-MMP15 or oe-LINC00482 + oe-FOXA1 + sh-MMP15 resulted in reduction of TNF-α, IL-1β, and IL-6 (*p* < 0.05) (Figure 5C). Meanwhile, tube formation was also found to be suppressed following the treatment with sh-MMP15 or oe-LINC00482 + oe-FOXA1 + sh-MMP15 (*p* < 0.05) (Figure 5D), and accompanied with reduced expression of VEGF and NF-κB (*p* < 0.05) (Figure 5E). The results of the aforementioned experiments suggested that silencing LINC00482 inhibited inflammation and angiogenesis through the down-regulating MMP-15 *via* FOXA1 recruitment in bladder cancer.

### Silencing LINC00482 suppresses progression of bladder cancer *in vivo*

**Figure 6:**
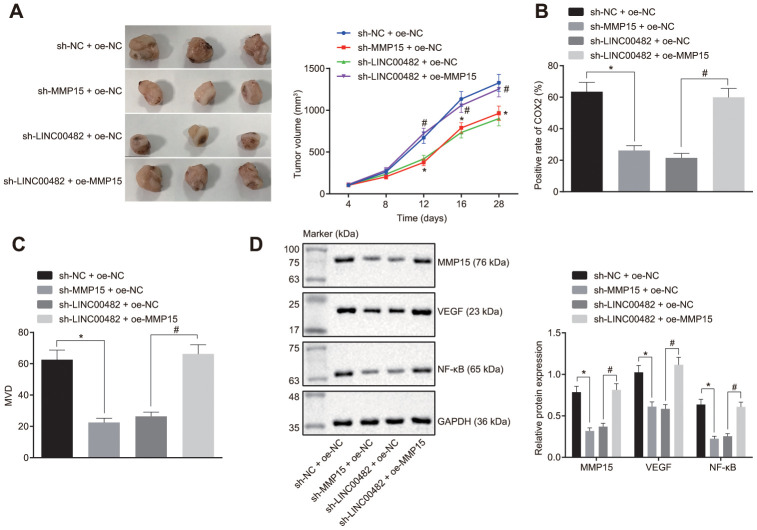
**Silencing LINC00482 suppresses progression of bladder cancer *in vivo.*** (**A**) Tumor volume in each group after injection of sh-MMP15, sh-LINC00482 and oe-MMP15; (**B**) immunohistochemistry of COX-2; (**C**) immunohistochemistry of MVD; (**D**) the expression of MMP15, VEGF, and NF-κB detected by Western blot analysis. The measurement data were presented as mean ± standard deviations, n = 8. Differences between two groups were compared by unpaired t test. Repeated measurement analysis of variance was used to compare the proliferation ability at different time points, and Tukey’s was used for post-hoc tests. * *p* < 0.05 *vs.* the sh-NC + oe-NC group; # *p* < 0.05 *vs*. the sh-LINC00482 + oe-NC group.

Additionally, tumor xenografts were transplanted in nude mice to study the *in vitro* findings. Difference of tumor volume following various treatments could be seen on the 12^th^ day. The growth of the tumor significantly slowed down after the injection with cells expressing sh-MMP15, whereas it was found to be accelerated following the treatment with oe-MMP15 (*p* < 0.05) (Figure 6A). Furthermore, according to immunohistochemistry studies (Figure 6B, 6C), treatment with sh-MMP15 decreased the amount of COX-2 positive cells and microvessel density (MVD), whereas the treatment with sh-LINC00482 + oe-MMP15 increased the amount of COX-2 positive cells and MVD when compared with sh-LINC00482 + oe-NC (*p* < 0.05). Additionally, the Western blot analysis indicated that the protein expression of MMP15, VEGF, and NF-κB were decreased in mice injected with sh-MMP15-treated cells, although sh-LINC00482 + oe-MMP15 treatment resulted in increased expression of MMP15, VEGF, and NF-κB when compared with sh-LINC00482 + oe-NC (*p* < 0.05) (Figure 6D). The above results established that silencing LINC00482 expression could potentially inhibit inflammation and angiogenesis *in vivo*, while overexpression of MMP15 could reverse this phenomenon.

## DISCUSSION

**Figure 7:**
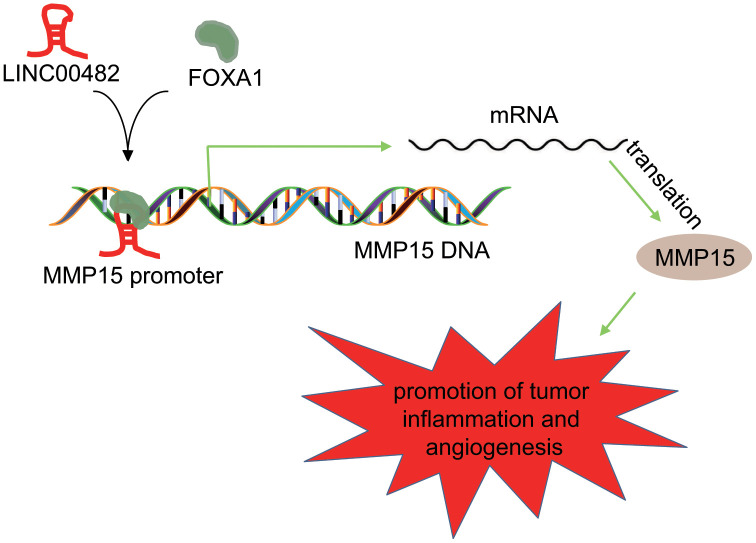
**Schematic diagram of the regulatory role of LINC00482 in bladder cancer.** LINC00482 was highly expressed in bladder cancer tissues and cells, while silencing LINC00482 could inhibit inflammation and angiogenesis through down-regulation of MMP-15 *via* recruiting FOXA1.

Characterized by a high incidence and recurrence rate, bladder cancer is considered to be the most common malignant cancer of urinary system [15]. The current protocol for treatment still fails to overcome this challenge effectively. Therefore, it is imperative to find effective bladder cancer biomarkers for early diagnosis, progression, and prognosis of this disease. LncRNAs have been established to play important roles in many oncogenic events in genitourinary diseases, including androgen receptor signaling pathway in prostate cancer, hypoxia-inducible factor regulatory network in renal cell carcinoma and tumor invasion in bladder cancer, as well as other underlying molecular mechanisms involved in survival and proliferation [16]. For instance, LINC00978 promoted the progress of bladder cancer through interacting with miR-4288, which indicates that LINC00978 might be a potential therapeutic target for bladder cancer treatment [17]. Based on the results of the bioinformatics analysis, LINC00482, a new-found lncRNA, was suggested to be highly expressed in bladder cancer tissues and cells. However, no data is available regarding the biological roles of LINC00482 in bladder cancer. In the current study, our findings suggested that silencing LINC00482 could potentially inhibit the proliferation, migration, invasion of bladder cancer cells through the up-regulation of MMP15 *via* recruiting FOXA1 (Figure 7).

We initially illustrated that LINC00482 was highly expressed in bladder cancer tissues and cells, and that the silencing LINC00482 could suppress the development and progression of bladder cancer. LncRNAs are transcripts with over 200 nucleotides in length and with low or no protein-coding potential and they also exert important functions in cancer signal transduction pathways *via* the interaction with proteins, lipids, RNA as well as DNA [7, 18]. Similarly, previous study revealed that down-regulating LincAC114812.8 in bladder cancer could significantly inhibit the proliferation, migration and invasion of bladder cancer cells both *in vitro* and *in vivo* [10]. A different study also confirmed that silencing LINC00319 suppressed the bladder cancer cell proliferation and invasiveness [19]. These studies reported that the down-regulation of LINCRNA PART1 could inhibit the cell proliferation and induce apoptosis in bladder cancer [20]. MMPs belong to a class of zinc-dependent extracellular matrix proteins and participate in cell invasion and metastasis as well as the carcinogenesis process [21]. The data from the current study showed that MMP15 expression was significantly increased in bladder cancer tissues and cells. Whereas, the other study established that MMP15 was found highly expressed in gastric cancer leading to poor prognosis in patients with gastric cancer [22].

Additionally, our results revealed that LINC00482 up-regulated the expression of MMP15 by recruiting FOXA1. Moreover, we found that silencing LINC00482 inhibited inflammation and angiogenesis in bladder cancer through the down-regulation of MMP-15 by targeting FOXA1, along with decreased levels of TNF-α, IL-1β, and IL-6 as well as the expression of VEGF and NF-κB. FOX transcription factor superfamily has been established to be differentially expressed in many subtypes of bladder cancer, and FOXA protein family plays crucial roles in the development of the embryonic bladder [23]. Empirical evidence elaborated that loss of FOXA1 was an independent indicator of reduced overall survival in bladder cancer patients [13]. Highly expressed MMP15 was also observed in non-small cell lung cancer, promoting angiogenesis and tumor progression [24]. Furthermore, previous studies have highlighted the important roles of lncRNAs involved in inflammation and angiogenesis [25, 26]. For example, decreased lncRNA TINCR has been shown to suppress the proinflammatory response through the downregulation of IL-6, TNF-α and CXCL1 [27]. Similarly, decreased LINC00482 in the present study also suppressed inflammation in bladder cancer by inhibiting the secretion of pro-inflammatory factors. Additionally, up-regulation of lncRNA ANRIL promoted angiogenesis of diabetes mellitus combined with cerebral infarction, as shown by increased expression of VEGF and NF-κB [28]. Partially in accordance to the present study, Jiang et al. showed that lncRNA SNHG15 could accelerate the progression of colon cancer *via* interacting with transcription factor Slug [29]. Finally, tumor xenograft in nude mice was conducted in our study, where silencing LINC00482 proved to inhibit inflammation and angiogenesis in bladder cancer *in vivo*.

The current study has indicated that LINC00482 could function as an oncogene and bind to the transcription factor FOXA1. Based on our *in vitro* and *in vivo* results, we conclude that silencing LINC00482 could down-regulate the expression of MMP15 *via* targeting FOXA1, which results in the inhibition of proliferation, migration, invasion of bladder cancer. In conclusion, our research highlighted the potential of LINC00482 as a novel therapeutic target for bladder cancer treatment. However, further investigations are needed to better understand by which mechanism LINC00482 initiates and regulates the onset of bladder cancer.

## MATERIALS AND METHODS

### Bioinformatics analysis

The Gene Expression Omnibus (GEO) database (http://www.ncbi.nlm.nih.gov/geo) was used to obtain the microarray data of bladder cancer-related expression profiling and the non-coding RNA profiling. The R package limma [30] was employed to analyze the microarray data to screen for differentially expressed lncRNA and mRNA in bladder cancer. After obtaining the largest values for logFC of lncRNA, the LncMAP (http://bio-bigdata.hrbmu.edu.cn/LncMAP/index.jsp) database was applied to predict the LncRNA-TF-Gene triplet. The candidate transcription factors were determined according to the number of triplets formed by each candidate transcription factor and related publications. Furthermore the Jvenn (http://jvenn.toulouse.inra.fr/app/example.html) was employed to compare the triplets of target genes and differential genes in GSE61615 to screen target genes. TF-genes with highest rewiring score were chosen as candidate genes for further studies.

### Sample collection

From January 2015 to January 2018, 84 patients with bladder cancer who underwent surgery in the First Hospital of Jilin University were enrolled for the present study. The subjects comprised of 56 male patients and 28 female patients aged between 38-88 years (mean age was 62.42 ± 8.41 years), as shown in Table 2. All patients in the study only received surgery.

The histopathological grade was determined according to the World Health Organization (WHO) classification criteria in 1999 [31], and the pathological staging was based on the TNM standards proposed by International Union Control Cancer (IUCC) in 2006 [32]. Patients were included if: 1, they were pathologically and genetically diagnosed bladder cancer; 2, they had no other digestive system tumors or tumor history; 3, they never received chemotherapy or radiation before the operation. They were excluded if: 1, they had severe functional impairments of heart, liver, kidney and other vital organs; 2, they had history of autoimmune disease; 3, they had chronic or acute infectious disease. All samples were processed within 30 min after resection and treated uniformly after collection. The study was approved by Ethics Committees of the First Hospital of Jilin University and written informed consents were obtained from all patients.

### Cell culture and transfection

Four human bladder cancer cell lines, HT-1376, T24, 5637(HTB-9), and J82 as well as human normal bladder epithelial cells (SV-HUC-1) were purchased from the cell bank of Cancer Research Institute of Central South University (Changsha, Hunan, China). The RT-qPCR analysis was employed to detect the expression of LINC00482 in the four human bladder cancer cell lines, out of which the cell line with the highest LINC00482 expression level was selected for subsequent experiments. The selected cells were further cultured in Roswell Park Memorial Institute 1640 (RPMI 1640) medium which contained 10% serum in a 37° C incubator with 5% CO_2_. When the cells reached about 80-90% of confluence, cells were passaged with fresh medium.

According to the known LINC00482, FOXA1 and MMP15 sequences in NCBI, LINC00482-ASO, the overexpression and NC-ASO plasmids were constructed by Sangon Biotech Co., Ltd. (Shanghai, China). Cells were then transfected with the following plasmids: LINC00482-ASO, oe-LINC00482, sh-FOXA1, oe-FOXA1, and sh-MMP15, as well as their corresponding controls. Cells in the log phase were trypsinized and seeded into 24-well plates to grow into a monolayer. Furthermore, the medium was then removed and cells were transfected in accordance to the instructions of Lipofectmine 2000. Fresh complete medium was added after transfection for 8 h and the cells were subsequently collected after 36-48 h for further experiments. Lipofectamine RNAimax (Invitrogen, Life Technology, USA) was used to transfect ASO to achieve a final concentration of 25 pmol. The subsequent procedures were the same as previously described.

### RT-qPCR

The total RNA was extracted from the tissues using Trizol whereas the total RNA from the cells was isolated in accordance with miRNeasy Mini Kit (217004, QIAGEN, Germany). The primers were synthesized by the Takara Company (Dalian, China) (Table 3). Subsequently the PrimeScript RT kit (RR036A, Takara) was used to reversely transcribe RNA into cDNA. Furthermore, Fluorescent quantitative PCR was performed with SYBR® Premix Ex TaqTM II (RR820A, TaKaRa). The real-time fluorescence quantitative PCR was detected by an ABI7500 quantitative PCR machine (7500, Applied Biosystems, 850 Lincoln Centre Drive Foster City, CA 94404, USA). The glyceraldehyde-3-phosphate dehydrogenase (GAPDH) was further used as internal control, and the relative expression levels of LINC00482, FOXA1, and MMP15 was analyzed by employing the 2^-ΔΔCT^ method: ΔΔCt = ΔCt _experimental group_ - ΔCt _normal group_, ΔCt = Ct _(target gene)_ - Ct _(internal control)_, relative transcription level = 2^-ΔΔCT^. To collect sufficient variable data the experiments were repeated three times.

**Table 3:** Primer sequences.

	**Primer sequences**
LINC00482	F: 5'-AGGGGTAACCTACCGGGAAA-3'
	R: 5'-CTTGGCCAGAGCTCCAGAAG-3'
FOXA1	F: 5'-GGGCGGTTCGGAGTTGA-3'
	R: 5'-CGAGGAGGACATGAGGTTGTTG-3'
MMP15	F: 5'-CATGCGTTCCGCCCAGAT-3'
	R: 5'-CCGCAGGTTGGCTTTCAC-3'
GAPDH	F: 5'-CCATGTTCGTCATGGGTGTGAACCA-3'
	R: 5'-GCCAGTAGAGGCAGGGATGATGTTC-3'

Note: F: forward; R: reverse.

### Western blot analysis

The total protein from the cells was extracted using the Radio-Immunoprecipitation Assay (RIPA) lysis buffer (R0010, Beijing Solarbio Science and Technology Co. Ltd, Beijing, China) supplemented with Phenylme thanesulfonyl fluoride (PMSF) and incubated on ice for 30 min, and centrifuged at 4° C for 10 min at 12,000 g. Protein (50 μg) was dissolved in 2× SDS loading buffer, boiled at 100° C for 5 min, separated by 10% sodium dodecyl sulfate polyacrylamide gel electrophoresis (SDS-PAGE) gel. After that, the proteins were transferred to poly vinylidene fluoride (PVDF) membrane which sealed with 5% skim milk powder at room temperature for 1 h. PVDF membrane was incubated overnight at 4° C with the following diluted primary antibodies: FOXA1 (1 μg/ml, ab23738), MMP15 (1: 300, ab135562), Ki67 (1 μg/ml, ab16667), MMP-2 (1 μg/ml, ab37150), VEGF (1: 2000, ab32152), NF-κBp105 (1: 5000, ab32360), GAPDH (1: 5000, ab8245 (all of the above antibodies were purchased from Abcam (Cambridge, UK)). The next day membrane was incubated with 1: 1000 diluted HRP-labeled secondary antibody goat anti-mouse IgG antibody (HA1003, Shanghai Yanhui Biotechnology Co., Ltd. Shanghai, China) for 1 h and reacted with enhanced chemiluminescence (ECL) solution (ECL808-25, Biomet, USA) for 1 min at room temperature for development. With GAPDH as an internal reference, the relative expression of protein was determined by the ratio of the gray value of the target band to the internal reference band. The experiment was repeated for three times.

### Dual-luciferase reporter gene assay

The target genes of LINC00482 were predicted by biological prediction website. The promoter region of MMP15 was inserted into pGL3 vector (Promega) to get recombinant plasmid, pGL3-MMP15. HEK293T cells were seeded in 24-well plate with a cell density of 3 × 10^4^/well. pGL3-MMP15 was co-transfected with oe-LINC00482 and LINC00482-ASO respectively with Rellina plasmid as reference. After transfection for 48 h, luciferase reporter gene was detected by dual luciferase reporter gene analysis system (Promega, Madison, WI, USA).

Through the analysis of UCSC (http://genome.ucsc.edu/) and JASPAR (http://jaspar.genereg.net/), it was found that FOXA1 protein was most likely to bind to three sites of MMP15 DNA. The recombinant luciferase reporter vector with truncated or mutated binding sites was co-transfected with FOXA1 expression vector into HT-1376 cells for dual luciferase reporter gene assay to verify the specific site of FOXA1 protein binding MMP15 DNA promoter region.

### RNA-FISH assay

The FISH assay was applied to study the subcellular localization of LINC00482 in bladder cancer cells in accordance to the instructions of the Ribo^TM^ LncRNA FISH Probe Mix (Red) (Ribobio, Guangzhou, China). Briefly, the cells were inoculated in the 24-well plate at a density of 6 × 10^4^ cells/well. Then the cells were subjected to incubation with 250 μL pre-hybridization buffer and then 250 μL FISH probe (300 ng/mL) in hybridization buffer at 42° C overnight. The air-dried slide was mounted with reagent with 4’, 6-Diamidino-2-Phenylindole for detection. Finally, 5 different fields of view were randomly selected under the fluorescence microscope (Olympus, Japan) for observation and photography.

### RIP assay

The cells were lysed and collected, and then the binding between LINC00482 and FOXA1 was detected by RIP kit (Millipore, Corp, Billerica, MA, USA) [33]. In brief, an aliquot of cell extract was saved as input and the rest was incubated with antibodies and magnetic beads. The magnetic bead antibody complex was resuspended in 900 μL RIP Wash Buffer after washing. The sample was placed on the magnet to collect the magnetic bead-protein complex. RNA was extracted and RT-qPCR was performed to detect LINC00482 and MMP15. The antibody used in RIP was FOXA1 (1 g/ml, ab23738, Cambridge, UK), along with IgG (1:100, ab172730, Cambridge, UK) as the negative control.

### ChIP assay

Cells were extracted, cross-linked by 16% formaldehyde, lysed by cell lysate and ultrasonicated, and then added with FOXA1 antibody to incubate overnight. Then, the magnetic beads were added to capture the protein DNA binding complex. After washing, 5 mmol/L NaCl was added for decrosslinking. Subsequently, DNA was recovered, and the binding of LINC00482 and MMP15 promoter in the complex was detected by RT-qPCR.

A primer (654-664 bp, 1319 bp from the transcription start site [TSS]) containing FOXA1 binding site 2 to MMP15 DNA promoter was designed (F: 5'-AGAGAAAATCACCAGACTTGACCGACC-3', R: 5'-GACAAGACCCCATCTGTACAAAGAATA-3') and the length of amplification product was 300 bp, which included the sequence of site 2 containing FOXA1 binding to MMP15 DNA promoter (654-664bp). Another primer different from the MMP15 DNA promoter region was designed to serve as a negative control (F: 5'-GTCCAGCATCAGTTTTCTCC-3', R: 5'-GATTTCTCTGTCCATCACCA-3') for the site 2 primer. The amplification product was measured to be 354 bp long and 4003 bp away from the TSS. Furthermore, the purified DNA fragment was used as the amplification template, and site 2 primer and Distal primer (control) were used to conduct the RT-qPCR experiment to verify whether site 2 of MMP15 DNA was the binding site of the transcription factor FOXA1.

Following the silencing of LINC00482, samples containing purified DNA fragments were isolated for the ChIP experiment (following the same method mentioned above). The NC-ASO group was used as the control group, the enrichment of MMP15 promoter site 2 bound to FOXA1 antibody was detected.

### ELISA

The cell supernatant was collected for the detection of TNF-α, IL-1β, and IL-6 which was achieved by using the corresponding ELISA kits (TNF-α, IL-6, IL-10 number: 69-25328, 69-40133, 69-98105, Wuhan Merck, Wuhan, China). The experiment was repeated three times to obtain sufficient and variable data.

### Tube formation experiment

Human vein endothelial cells HUVECs (INS-1, Shanghai Zishi Biotechnology Co., Ltd., Shanghai, China) were regularly cultured and collected. The bladder cancer cell culture was then centrifuged at 3000 r/min for 3 min with the supernatant collected for the subsequent resuspension of the HUVECs. Following this the cells were inoculated in Matrigel coated 24-well plate with a density of 1 × 10^5^/well for 24 h at 37° C and atmosphere of 5% CO_2_. An inverted microscope was used to record the results from three randomly chosen fields.

### Transwell assay

The Transwell chamber was coated with Matrigel. Cells were incubated in a serum-free medium, digested, and then resuspended in a medium containing 5% serum. The resultant cell suspension (150 μl) was added to the Transwell chamber where medium containing 20% serum (600-700 ul) were added to 24-well plates with medium containing 20% serum (600-700 ul) for incubation for 48 h. The chamber was further washed with PBS, fixed with 100% methanol for 15-20 min, and stained with 0.1% crystal violet for 15-20 min. The invading cells were observed and photographed under a microscope.

### Xenograft tumor in nude mice

A total of 32 BALB/c-nu/nu male nude mice (6-8 weeks old, Shanghai SLAC Laboratory Animal Co., Ltd, Shanghai, China) at SPF grade were selected. Stable transfected cell lines HT-1376 of sh-NC + oe-NC group, sh-MMP15 + oe-NC, sh-LINC00482 + oe-NC, and sh-LINC00482 + oe-MMP15 were established. Bladder cancer cells in logarithmic growth stage were taken with the cell density adjusted into 2.5 × 10^7^/mL. Stably transfected cell line HT-1376 (200 μL) were subcutaneously inoculated in the nape of nude mice which were further raised. The tumor volume was measured once a week, and the maximum diameter (a) and minimum diameter (b) of the transplanted tumor nodules in nude mice were measured with vernier caliper, and the tumor volume (V) was calculated with the formula of V = 1/2 × a × b^2^. The mice were sacrificed on the 28^th^ day, and the tumors were dissected and removed. Three tumors in each group were collected and made into paraffin sections.

### Immunohistochemistry

The paraffin embedded sections were dewaxed, dehydrated, soaked in 3% H_2_O_2_ for 10 min, underwent antigen-retrieval with high pressure for 90 s, and washed with PBS. Next the sections were incubated with 5% BSA at 37° C for 30 min, then probed with rabbit anti-mouse polyclonal antibody MMP15 (1: 300, ab135562, Cambridge, UK), polyclonal antibody COX-2 (1: 1000, ab15191, Cambridge, UK) and polyclonal antibody CD34 (1: 4000, ab81289, Cambridge, UK) overnight at 4° C. Following the subsequent washing with PBS for 2 min, the sections were incubated with 50 μl Biotinylated goat anti-rabbit IgG (1: 100, ab172730, Cambridge, UK) at 37° C for 30 min. SAB working solution was added, and the sections were stained using Diaminobenzidine (DAB), then counterstained by hematoxylin for 5 min, washed, dehydrated, cleaned, sealed and finally observed under the microscope. PBS was replaced as the primary antibody for negative control. The immunohistochemistry analysis [34] revealed that more that 25% area was stained brownish-yellow in the positive cells and the staining was chiefly located in the cytoplasm, cell membrane and the endothelial of the tube. The microvessel density (MVD) count was calculated based on the CD34 positive cells, clusters or individual endothelial cells or with clear boundaries with adjacent tumor cells, microtubules and its surrounding connective tissues. If the structure was not connected, it was counted as a tube. The positive expressions of MMP15, COX-2 and MVD were observed in 5 randomly selected fields under a high magnification microscope. The experiments were repeated three times to obtain sufficient and variable data.

### Statistical analysis

Study data were analyzed using the SPSS 22.0 (IBM, Armonk, NY, USA) statistical software. The quantitative data were presented as mean ± standard deviations. Data conforming to normal distribution and equal of variance within a group were analyzed by paired *t*-test. Meanwhile the differences between two groups were compared by employing the unpaired *t*-test. Comparisons among multiple groups were analyzed by one-way analysis of variance, followed by a Tukey’s post-hoc test. Repeated measurement analysis of variance was used to compare the proliferation ability at different time points, and Tukey’s was used for post-hoc tests. Furthermore, Pearson correlation coefficient was used to analyze the correlation between LINC00482 and MMP15 expression in bladder cancer tissues. *p* < 0.05 was considered statistically significant.
